# Equity Beyond Entry: A Capability Approach to Understanding Widening Participation in Medical Education

**DOI:** 10.5334/pme.2071

**Published:** 2025-11-24

**Authors:** Ashley Simpson, David Hope, Jeni Harden, Lorna Marson, Victoria Tallentire

**Affiliations:** 1 The University of Edinburgh, UK; 2 NHS Lothian Medical Education Directorate, UK; 3 The University of Edinburgh, UK

## Abstract

**Introduction:**

Widening participation aims to promote equity in medical education and build a more diverse healthcare workforce. Despite ongoing efforts, systemic inequities persist in shaping students’ trajectories. This study explores how widening participation students experience medical education, examining the factors that enable or constrain their success across the continuum of entry and progression. It offers a novel contribution by applying the capability approach to explore the conditions that influence students’ freedoms to pursue valued outcomes.

**Methods:**

This qualitative study employed semi-structured interviews with 16 medical students from widening participation backgrounds in Scotland. Data were analysed using reflexive thematic analysis and interpreted through the capability approach, which considers individuals’ freedoms to pursue outcomes they value. Central to this are personal, sociocultural, and institutional conversion factors that determine whether individuals can transform available resources into genuine opportunities.

**Results:**

Participants described persistent capability constraints. Early aspirations were undermined by discouragement and low expectations from teachers and advisers. Educational disadvantage hindered preparation for entry. Once admitted, financial insecurity, opaque support systems, cultural exclusion, and limited career guidance restricted capabilities. Privilege functioned as an enabling conversion factor but was unevenly distributed. Structural supports were limited, with enabling factors more often linked to individual resilience than to institutional provision.

**Conclusion:**

Achieving equity in medical education requires more than just widening access; it demands attention to systemic barriers that shape students’ experiences and outcomes. Applying the capability approach highlights the need to transform institutional conditions that influence students’ freedoms to succeed, offering both theoretical and practical insights for reform.

## Introduction

Transforming higher education from an exclusive privilege to an accessible resource for all, widening participation policies embody the principles of social justice. By dismantling barriers, raising aspirations, and creating equitable pathways for individuals from historically underrepresented groups, these policies strive to foster equity, diversity, and inclusion across the educational landscape [1]. In the UK, widening participation refers to coordinated strategies that improve access to and success within higher education for students who are underrepresented due to socioeconomic, geographic, or educational disadvantage. The ultimate goal is to establish an educational system in which sociodemographic background does not determine who accesses or succeeds at university and beyond [2].

While the terms widening access and widening participation are often used interchangeably, they reflect different emphases. Widening access primarily focuses on initiatives to increase entry, such as outreach programmes, alternative admissions pathways, and contextual offers. In contrast, widening participation encompasses the entire student lifecycle, addressing entry, retention, progression, and success. This distinction is critical in medical education, where access schemes may increase entry but fail to address the structural and cultural challenges, as well as the financial inequities, that can persist or emerge after admission. This study adopts the term widening participation to capture a holistic orientation towards equity throughout medical education.

In the UK, the commitment to widening participation is underpinned not only by principles of social justice, but by practical and professional imperatives. These include the need to expand and diversify the medical workforce, alongside evidence that a workforce that reflects the demographics of the population will improve patient care [3,4] and reduce healthcare inequalities [5,6]. Consequently, policymakers have positioned widening participation as both a moral and pragmatic endeavour – a means of redressing inequity and addressing local workforce shortages by encouraging students from deprived or rural backgrounds to enter and remain in the medical profession [7].

Despite over 50 years of targeted initiatives [8], individuals from low socioeconomic backgrounds remain underrepresented in medical education [9,10]. This enduring disparity reflects multiple and intersecting barriers, including low expectations of success [11,12,13], lack of support and encouragement [14,15,16], limited knowledge of application processes [11,13,15,16,17,18], difficulty accessing work experience [15,16,18], and financial concerns [11,15]. Increasingly, research demonstrates that inequities extend beyond admission, shaping students’ experiences throughout training [19,20]. Students from underrepresented backgrounds experience feelings of alienation [21,22] and isolation [23,24], difficulties developing social networks [25] and tensions between professional and sociocultural identities [22,23,26,27,28]. Financial constraints further heighten stress and reduce wellbeing [29,30]. Without addressing these challenges beyond entry, the transformative intent of widening participation risks being undermined.

**Figure 1:**
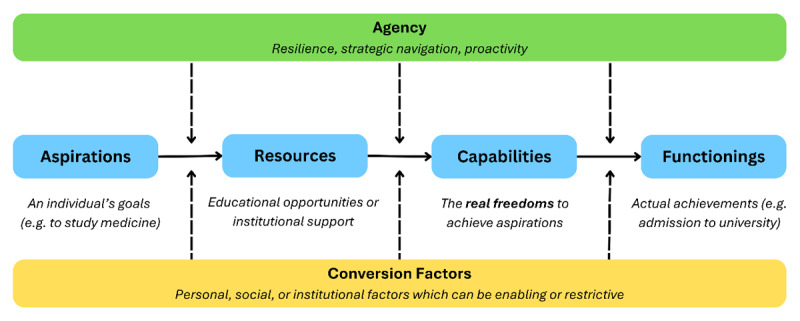
**The capability approach applied in medical education**. Building on Sen’s capability approach, the figure illustrates how individual aspirations interact with available resources to form capabilities (understood as the freedoms to achieve valued goals), which may then be realised as functionings (actual achievements). This process is mediated by conversion factors (personal, social, and institutional conditions that enable or constrain) and by agency (the capacity to navigate structures with resilience and strategic action).

Traditionally, success in widening participation has been measured by entry rates and academic outcomes. Yet, genuine equity demands attention to how institutional and social structures shape students’ capacity to thrive once admitted. Developed by Amartya Sen, the capability approach reframes equity as a matter of real freedoms rather than formal opportunities [31]. It recognises that individuals differ in their ability to convert educational resources into meaningful advantage, depending on personal, social, and institutional contexts [32,33]. Within this framework, *capabilities* denote the genuine freedoms or opportunities a person has to convert resources into valued outcomes, while *functionings* are the achievements realised through those opportunities. Central to this model are *conversion factors* – personal, social, or institutional influences that determine how individuals translate aspirations into achievements. (Figure 1, p. 3). The capability approach thus provides a powerful lens for this inquiry, shifting the focus from formal access to what individuals are genuinely able to achieve within specific educational contexts.

By employing the capability approach, this study offers an alternative to deficit-based interpretations – often grounded in Bourdieu’s concept of capital – that dominate much of the widening participation literature. It examines how educational environments enable or constrain what students can actually do or become – their *real* freedoms to pursue valued outcomes. Critical to this approach is agency: the capacity of individuals to make choices and act in pursuit of their goals. Accordingly, the study reframes widening participation not simply as a matter of access or attainment, but as equity in capabilities – what valued outcomes students are genuinely able to achieve within the structural realities of medical education.

Guided by this framing, the study asks: In what ways do personal, social, and institutional conversion factors enable or constrain the capabilities of widening participation medical students as they navigate undergraduate medical education?

### Research Context

This study is situated in Scotland, where widening participation policies are nationally coordinated but locally implemented. Here, the term widening participation encompasses a broad range of circumstances associated with underrepresentation in higher education, including, for example, coming from a low-income household, eligibility for free school meals, attending a school with a low progression rate into higher education, holding refugee or asylum seeker status, having experience of the care system, or being estranged from family. While broader UK literature often includes minoritised ethnic groups under the widening participation umbrella, this is not a standalone criterion in Scotland. However, recognising the intersectional nature of disadvantage, this study includes individuals from minority ethnic backgrounds.

Across the UK, widening participation initiatives typically involve targeted outreach programmes, contextual admissions, foundation or gateway programmes, and additional academic and pastoral support to improve access and retention. How these initiatives are defined, prioritised, and operationalised varies between institutions [34].

While the context of this study is specific to Scottish medical education, the structural barriers identified – financial precarity, cultural exclusion, and the hidden curricula – resonate across international contexts. The findings, therefore, offer broader insights for global efforts to diversify the medical profession and create environments in which all students can thrive.

## Methods

### Research paradigm

We adopted a qualitative design informed by a constructivist paradigm, which views reality as socially constructed and shaped by individual experiences [35]. We co-created knowledge with participants through interaction, interpretation, and reflexivity, recognising how context, social structures, and individual perspectives influence students’ experiences. We chose this paradigm to explore the interplay between agency and structural constraint, which is central to both widening participation and the capability approach. Although critical paradigms more explicitly foreground power and inequality, constructivism enabled us to engage with both subjective meaning-making and broader institutional context. Throughout the study, we examined how participants’ narratives reflected and negotiated structural realities.

### Researcher reflexivity and positionality

Consistent with reflexive thematic analysis, we recognised our active role in shaping knowledge. The lead researcher (AS), a senior resident doctor and postgraduate research student, is a first-generation university graduate from a socioeconomically deprived background. She conducted all interviews and led the analysis. Her shared background with participants enhanced rapport and interpretive depth, but also introduced potential bias, which she addressed through reflexive journaling and regular supervisory discussions.

The supervisory team comprised: DH, a Professor of Psychometrics; JH, a Professor of Social Science in Medicine; LM, a Professor of Transplant Surgery and former Dean of Admissions and Widening Participation; and VT, a Consultant Physician and Associate Director of Medical Education for Scholarship with no prior experience in widening participation. Their diverse disciplinary and professional perspectives challenged assumptions, refined interpretations, and strengthened analytic depth.

### Participants

#### Recruitment

We recruited participants through email invitation and posters distributed across four Scottish medical schools (Aberdeen, Dundee, Edinburgh, and Glasgow), three of which host gateway programmes for students from widening participation backgrounds. Eligible participants self-identified as final-year students from widening participation backgrounds. We outlined each university’s widening participation criteria during recruitment, but did not request proof of eligibility. This ethically informed decision was favoured over targeted recruitment to avoid singling out students or provoking identity discomfort among those who met criteria but did not identify with the label. Seventeen students responded to the invitation, and sixteen consented to participate. We informed participants of the study’s aims and procedures, obtained written consent, and reminded them that participation was voluntary and that they could withdraw at any time before analysis.

#### Demographics

The final sample included students with varied entry routes, ages, and experiences. Nine entered medicine via standard entry programmes, four through gateway programmes, and three via postgraduate pathways. Participants ranged in age from 23 to 34, with the majority in their mid-twenties – consistent with UK medical graduates. Thirteen identified as female (81%), and three as male (19%). Most participants (n = 13, 81%) identified as socioeconomically disadvantaged and/or from low progression secondary schools. Less common experiences included estrangement from family (n = 1), caring responsibilities (n = 1), and being a first-generation immigrant (n = 1), aligning proportionally with Scotland’s widening participation profile.

Twelve participants (75%) self-identified as White British, and four (25%) as racially or ethnically minoritised. Although we did not explicitly ask about disability, two participants disclosed relevant experiences during interviews. To protect anonymity in this small sample, we have chosen not to associate individual participants with their specific widening participation criteria or background characteristics. The final sample of sixteen participants (Appendix 1) was judged to hold sufficient information power to address the study aim, providing rich, diverse accounts suitable for in-depth reflexive thematic analysis.

### Data collection

Between March and June 2024, after final examinations, AS conducted sixteen semi-structured interviews in person or via secure online platforms, according to participant preference. We used semi-structured interviews because they provided the flexibility to explore individual meanings while maintaining a consistent focus on key topics. This approach aligned with our constructivist paradigm and reflexive thematic analysis, enabling participants to shape the direction of discussion and articulate experiences in their own terms. It also allowed us to probe how personal, social, and institutional factors influenced students’ capabilities and sense of agency, consistent with the capability approach.

We developed the interview guide (Appendix 2) from the existing literature on widening participation and medical student experience, drawing on studies addressing access barriers, identity, belonging, and structural inequality. We piloted the guide with two widening participation students not involved in the main study, leading to minor revisions that clarified wording, emphasised structural influences, and reordered questions to build rapport before exploring identity and belonging.

AS audio-recorded, transcribed and anonymised all interviews, assigning pseudonyms chosen or approved by participants. A master key linking pseudonyms to real names was encrypted and stored separately.

We invited participants to review their transcripts and redact data before analysis to maintain agency over their narratives. We did not conduct member checking, as this is not typically aligned with the epistemological foundations of reflexive thematic analysis.

AS approached interviews reflexively, aware that her dual identity as a widening participation graduate and medical educator could feel both familiar and authoritative. She mitigated power imbalances and created a safe, non-judgmental space for open dialogue.

### Data analysis

We analysed data using reflexive thematic analysis, as outlined by Braun and Clarke [36]. This approach, aligned with our constructivist paradigm, views meaning as co-constructed through interpretation and reflexivity and recognises the researcher’s active role in shaping, rather than discovering, themes.

We followed Braun and Clarke’s six phases: 1) data familiarisation; 2) initial coding; 3) developing preliminary themes; 4) reviewing and refining themes; 5) defining and naming themes; and 6) producing the final report. We moved iteratively across stages, guided by both the data and emerging theoretical insights.

We used the capability approach to foreground opportunity, constraint, agency, and valued outcomes. During coding and theme development, we examined how participants described the interplay of resources, conversion factors, capabilities, functionings, and agency, attending to latent meanings, social positioning, and structural contexts. This approach enabled us to move from descriptive codes (e.g., teacher discouragement, financial hardship) to interpretive themes (e.g., capability to aspire, capability to succeed), illustrating how structural and social conditions shaped students’ real freedoms. We retained and examined contradictory or divergent accounts as critical counterpoints, revealing the heterogeneity of experiences among widening participation students and the uneven operation of conversion factors across contexts.

### Ethical approval

We obtained ethical approval from the Medical Education Ethics Committee at the University of Edinburgh, with subsequent approval from other participating institutions.

## Results

This manuscript presents results through the lens of the capability approach, focusing on how social, institutional, and cultural conversion factors enable or constrain widening participation students’ capabilities, functionings, and agency throughout their educational journeys. By analysing participants’ accounts through this framework, the study reveals how inequity operates not as an isolated disadvantage but as a constrained conversion of opportunity into real freedoms and valued outcomes.

### The capability to aspire: *Recognition and misrecognition*

Aspiration is a socially situated capability shaped by recognition and institutional conversion factors. For many participants, schools did not cultivate this capability but constrained it, often through subtle or overt sociocultural bias. Teachers and careers advisers – key institutional gatekeepers – were frequently described as discouraging or dismissive when students expressed interest in medicine.

Elsie recalled:

“One teacher laughed when I told him that I wanted to do medicine. […] I just don’t think he believed I’d get the grades. I guess the school isn’t used to having high-achieving pupils.”

In capability terms, this reflects a failure of institutional recognition, which restricts the conversion of aspiration into action and reproduces social hierarchies about who medicine is ‘for.’

Leah’s experience with a careers adviser illustrated how classed assumptions narrowed aspirational horizons:

“I remember speaking to the careers adviser, and they were like, ‘Oh, you should be a secretary.’ […] There was no higher aspirations. There was no suggestion that you could go and do medicine. The people who mentioned it were very much talked out of it, ‘Don’t do that, that’s not for the people who go to this school.’”

Here, restrictive social conversion curtailed the capability to aspire by constraining what was imaginable, diverting students toward roles aligned with deficit-based assumptions.

Andrew described a broader culture of low expectations:

“I got the impression that the school only really cared about you not getting caught dealing class A drugs, getting someone pregnant, or stabbing someone. Anything other than that was kind of just a bonus.”

For participants, ambition often began in spaces where medicine was unacknowledged or discouraged. This institutional climate produced capability deprivation by undermining recognition as a precursor to aspiration. Students’ ability to resist these pressures represented agency under constraint—a survival strategy that should not be romanticised but understood as a response to structural misrecognition.

### The capability to achieve: *Educational disadvantage*

Although participants had gained entry to medical school, their accounts revealed stark inequities in educational foundations. From a capability perspective, this demonstrates that formal access to education is insufficient; what matters is the ability to convert resources into valued outcomes through enabling conversion factors.

Adam recalled:

“Most subjects, you’d have only one teacher who’s doing that subject. The reason that becomes a problem is that if that teacher is off for any reason […] you’re now left with no one to teach your class. […] You’re sitting in a classroom essentially learning nothing.”

Adam had formal access to schooling but lacked the conditions to convert that access into achievement; his real freedom to succeed was constrained by an environment that could not consistently support his learning.

Leah described how high staff turnover led to widespread academic failure:

“We had a new teacher every three weeks […] Myself and all the people who are in my A levels, we all failed. But that’s not because we’re not smart, it’s just that we were being taught the wrong curriculum.”

Her experience highlights that educational achievement is contingent on institutional conversion factors, not just individual effort. The capability approach exposes how structural deprivation is misread as individual deficit, reframing failure as a reflection of inequitable conditions rather than personal inadequacy.

For some, these deficits demanded self-directed learning. Katie explained:

“… for sixth year, I only had three classes a week, the rest of it was self-taught.”

Katie’s experience exemplifies how ‘resilience’ operates as a compensatory conversion factor, masking structural inequity. The burden of ‘working harder’ becomes a hidden requirement for success, unevenly distributed. From a capability standpoint, these inequities transfer the responsibility for achievement from the institution to the student, constraining real freedoms to achieve.

### The capability to belong: *Cultural and institutional exclusion*

Belonging is a socially and institutionally mediated capability—a freedom to participate and be recognised within a community. For many participants, belonging was unequally distributed through the operation of social and cultural conversion factors such as classed norms, linguistic hierarchies, and institutional cultures.

Elsie, who studied at a university close to home, described being mocked by her peers for her regional accent:

“… it was a lot harder to be taken seriously than some other people […] they would hear my thick Scottish accent and would all of a sudden just start laughing or trying to imitate.”

Though seemingly minor, such microaggressions revealed institutionalised forms of exclusion. For Elsie, language became a marker of difference, rendering her identity less legitimate and constraining her capability to belong. Despite studying in her hometown, she felt displaced:

“… although I was in my hometown, like, where I’m from, where I’ve always been from, I still felt like the minority. […] There weren’t that many people like me.”

Elsie was not marginalised by geography or academic ability, but by the dominant institutional culture in which access did not equate to inclusion.

Val described the cultural distance she felt in peer conversations:

“… when people start talking so casually about certain things that are very normal in their class, like, ‘Oh I went skiing’ or ‘I went on this holiday’ or ‘that gap year.’ I used to look at them like, ‘Wow, we have lived very different lives.’”

These everyday exchanges reinforced symbolic boundaries and the unequal distribution of social capital, shaping who could fully participate. Belonging thus emerges as a collective capability that depends on institutional conversion factors that affirm or marginalise identities.

### The capability to succeed: *Financial insecurity*

For many participants, success was constrained not by ability but by financial insecurity and inflexible institutional conversion factors. Although formally admitted, students’ real freedoms were curtailed by the need to work, lack of targeted support, and burdensome funding processes.

Nicole highlighted how institutional assumptions overlooked lived realities:

“… when I first failed [an exam], I had a one-on-one chat with my personal tutor and they basically said, ‘You should quit your job and focus on medicine’, […] They were like, ‘There’s different sorts of funding available’, but offered no direction towards it.”

This response reveals a key tension – access to resources does not equate to the capability or agency to use them effectively. From a capability perspective, institutional support must be both available and accessible. Without clear guidance, Nicole was left to manage structural pressures alone, while institutional assumptions framed financial stability as a personal issue.

Leah’s experience underscored how institutional responses to hardship were framed in ways that pathologised need:

“If you go to [the medical school] and say, ‘Oh, I’ve got financial stress,’ then they say, ‘Well, here’s a hardship fund,’ but actually, ‘For you to have the hardship fund, we’re gonna look at you with a fine-tooth comb. We’re gonna go through everything you’ve ever spent money on.’ That’s not a solution for me at all. […] I need the environment to be able to support myself. I don’t want support from anybody else. I don’t want a hardship fund. I don’t want a food bank. I don’t want a free pizza party. What I want is my timetable in advance. That’s a reasonable adjustment to me.”

The university’s response to Leah’s financial situation failed to address the structural barriers limiting her capability to succeed. The hardship fund was inaccessible due to its invasive process, failing as a conversion factor. Leah’s request for advanced timetabling, a genuine structural solution, would have enabled independent capability and agency. By offering only reactive, short-term solutions, the institution perpetuated deficit narratives and reinforced a sense of capability deprivation.

### The capability to advance: *Privilege and progression*

Although all students accessed the same curriculum, their actual freedom to convert opportunity into advancement varied according to their access to social, cultural, and academic capital. Privilege functioned as an enabling conversion factor, facilitating access to electives, research, and networks that advanced careers.

Ailish explained:

“I know a lot of people who’ve done [student selected components] because their dad knows someone and they were able to get a contact, […] that just wasn’t something that I would have. It was a network of people that I didn’t have any sort of way to tap into at all.”

Adam added:

“… because his dad is a doctor, a professor, he has four publications […] handed to him on a plate.”

Though capabilities were theoretically equal, Ailish and Adam’s functionings differed because enabling conversion factors were unequally distributed. While their friends effortlessly gained access to opportunities, Ailish and Adam had to navigate these barriers independently, relying on personal conversion factors, such as motivation and resilience, expending additional effort for the same outcome.

Freya reflected on confidence as a socially conditioned form of agency:

“I feel like maybe from my upbringing, in a sense, you just don’t ask for things. Like, you just get what you’re given. […] Whereas I know one of my quite good friends, she’s definitely emailed people outright, she’s a lot more confident to just ask if there’s any research that she could get involved with. Both her parents are university professors or academics.”

Confidence here emerges not as a personality trait but as a socially-generated capability, shaped by upbringing and expectation. The uneven distribution of networks, confidence, and insider knowledge demonstrates how privilege mediates students’ capabilities and agency to advance. From a capability standpoint, advancement depends not only on the availability of opportunities but also on the conversion factors that determine who can act upon them.

Taken together, these findings illustrate that widening participation in medical education extends far beyond entry. Across the five capabilities – to aspire, achieve, belong, succeed, and advance – students’ experiences reveal how personal, social, and institutional conversion factors interact to shape what they are genuinely able to do and become. While formal access opens the door, inequitable conversion conditions determine whether participation translates into real freedoms and valued outcomes. Agency often emerged as resistance under constraint rather than evidence of equal opportunity. These findings underscore that achieving equity in medical education requires transforming the structural and cultural conditions of medical training so that capability expansion becomes the true measure of success in widening participation.

## Discussion

### Reframing inequity through the capability approach

This study demonstrates that students from widening participation backgrounds face persistent structural barriers that hinder their ability to convert opportunities into genuine capabilities during their medical education. While previous research has identified similar inequities, the novelty of this study lies in applying the capability approach to reconceptualise these challenges as failures of institutional conversion factors that constrain students’ real freedoms to achieve valued outcomes.

By adopting this lens, we move beyond access-based definitions of equity to examine how opportunity is structured, distributed, and converted into real advantage. The capability approach exposes the limits of inclusion narratives that equate equity with access, instead foregrounding institutional responsibility for creating environments that expand capability. Rather than focusing on what widening participation students are perceived to lack, we foreground what institutions fail to meaningfully provide.

Our analysis extends previous applications of the capability approach, demonstrating its analytic power to uncover context-specific mechanisms of constraint and adaptation. Participants varied in how they negotiated structural barriers: some exercised their agency by actively engaging mentors or creating self-directed opportunities, while others were restricted by financial or cultural constraints. This variability highlights how capabilities are relational and contingent, shaped through interaction between agency and the conversion factors that enable or restrict it.

This framework also challenges the widespread valorisation of resilience. Within a capability perspective, resilience is better understood as agency under constraint – an adaptive, often exhausting response to capability deprivation rather than an index of strength. The extraordinary efforts students make to overcome institutional shortcomings both obscure systemic inequity and individualise the responsibility for success.

Overall, the capability approach provides a critical and theoretically coherent framework for understanding how students navigate unequal conditions. It shifts attention from individual attributes to the institutional and structural processes that expand or restrict capability, reframing equity as the expansion of students’ real freedoms and agency to thrive. This theoretical application constitutes the study’s primary contribution to medical education scholarship, demonstrating the utility of the capability approach as a framework for diagnosing and addressing persistent inequities in access, belonging, and progression. In doing so, this study extends the capability approach beyond its prior applications in health and higher education, demonstrating its explanatory power for understanding the structural dynamics of equity in medical training.

### Situating findings in an international context

Our findings contribute to global debates on equity in medical education by highlighting the limitations of access-focused models and the need for sustained structural support across the educational continuum. Participants emphasised that meaningful inclusion required not only gaining access to medical school, but having the institutional conversion factors – such as mentorship, financial stability, inclusive pedagogy, and predictable timetables – necessary to sustain participation and achievement. These insights are particularly relevant in contexts where widening participation continues to be evaluated through narrow metrics of enrolment rather than capability expansion.

The capability approach provides a comparative and transferable framework for assessing substantive equity across systems. By asking whether students have the real freedom and agency to achieve valued outcomes, it directs attention beyond formal opportunity to the conditions that determine whether those opportunities can be realised. Whether in quota-based or meritocratic systems, a capability-informed perspective redefines fairness not as equal access, but as the equal ability to convert access into opportunity.

This framing also offers a practical policy lens: equity efforts should be judged not by who enters medical school, but by who is supported to thrive, progress, and advance. Future research could explore how national contexts shape the conversion factors that constrain or enable underrepresented students, and how institutions can adapt their practices to expand students’ capabilities and agency. In this way, our study offers both a contextualised insight into Scottish medical education and a transferable conceptual contribution to international discussions on how medical schools can move from formal access to substantive equity.

### Institutional critique

Participants consistently identified institutional shortcomings in career guidance and financial support as significant barriers to equitable participation. While grounded in perceptions rather than auditable data, the consistency of these accounts suggests systemic weaknesses in how medical schools enable students to translate formal resources into real opportunities.

Financial support mechanisms were frequently described as opaque, bureaucratic, or stigmatising. Although hardship funds existed, their design and delivery failed as enabling conversion factors, undermined by intrusive eligibility checks and poor communication. In capability terms, financial resources were available but not accessible, thus were ineffective in enabling opportunities.

Similarly, students described a lack of inclusive and transparent career guidance. Those without inherited professional networks or insider knowledge often relied on informal advice or personal initiative to navigate complex postgraduate pathways, creating an uneven playing field for career development. This gap constrained students’ capability to advance, reinforcing existing inequalities and limiting agency.

These findings underscore the requirement for institutional resources that are not only available but genuinely accessible, navigable, and responsive to students’ lived realities. Future research should examine how institutional supports operate as enabling or restrictive conversion factors and identify strategies that transform institutional processes from nominal provision into genuine capability enhancement.

### Strengths and Limitations

#### Strengths

This multi-institutional qualitative study provides rich insight into how structural and institutional factors shape widening participation students’ educational trajectories. By explicitly applying the capability approach, our study offers a theoretically grounded lens that foregrounds institutional accountability rather than individual deficit. Exploring experiences across the full educational continuum enables a holistic understanding of how capabilities evolve, informing policy discussions aimed at sustained equity and structural reform in medical education.

#### Limitations

Self-identification as widening participation may have introduced selection bias, with students holding stronger views more likely to participate. The retrospective design raises potential recall and courtesy bias, as participants may have moderated criticism given the researcher’s institutional role. Furthermore, this study did not capture experiences of students who withdrew or did not complete their medical training, thereby potentially overlooking the most severe forms of capability restriction. Transferability is limited by the Scottish context and local definitions of widening participation. Future research should examine racialised, disabled, or other marginalised groups to capture the full spectrum of capability constraints and opportunities for expansion.

## Conclusion

Traditional measures of widening participation – such as admission and completion rates – offer only a partial view of equity. While these metrics assess entry and retention, they overlook whether students can effectively convert educational resources into valued outcomes. Using the capability approach, this study shows that students’ real freedoms to thrive depend not only on access but on the institutional and sociocultural conditions that shape capability and agency.

Reframing widening participation through institutional conversion factors rather than individual deficits provides a more justice-oriented understanding of equity. Structured mentorship, anticipatory timetabling, inclusive pedagogy, and accessible financial support emerged as practical strategies to reduce constraints, expand real freedoms, and promote equitable outcomes. As one participant reflected, “I need the environment to be able to support myself.” This sentiment encapsulates the study’s central insight: equity depends not on individual resilience but on environments that enable all students to act and achieve as valued members of the profession.

A capability-informed approach invites medical schools to move beyond symbolic inclusion towards structural transformation. By embedding equity in everyday practices and evaluating success by the freedoms students truly possess, medical education can progress from widening access to realising widening participation – building a more diverse and representative workforce equipped to meet the needs of an increasingly diverse society.

## Additional Files

The additional files for this article can be found as follows:

10.5334/pme.2071.s1 Appendix 1. Participant Demographics.

10.5334/pme.2071.s2 Appendix 2. Interview Guide.
