# Supplementary material for: Equity Beyond Entry: A Capability Approach to Understanding Widening Participation in Medical Education
Source: Perspect Med Educ. 2025 Nov 24;14(1):871–81. doi: 10.5334/pme.2071 (PMC12662159; doi:10.5334/pme.2071)
Supplement: Appendix 1. — Participant Demographics. [file pme-14-1-2071-s1.pdf]

Table 1 Participant demographics

Pseudonym	Age	Gender	Route of entry
Freya	24	F	Standard entry medicine
Lisa	24	F	Standard entry medicine
Adam	24	M	Standard entry medicine
Joanna	24	F	Standard entry medicine
Jack	24	M	Gateway programme
Ailish	24	F	Gateway programme
Julie	24	F	Standard entry medicine
Rosa	25	F	Gateway programme
Andrew	30	M	Postgraduate entry to medicine
Val	23	F	Standard entry medicine
Nicole	24	F	Standard entry medicine
Leah	27	F	Postgraduate entry to medicine
Katie	24	F	Standard entry medicine
Elsie	24	F	Standard entry medicine
Anna	24	F	Gateway programme
Jane	34	F	Postgraduate entry to medicine

In keeping with the ethical commitments of the study, we have chosen not to associate individual participants with their specific widening participation criteria or background characteristics. This decision was taken to protect anonymity and reduce the risk of indirect identification, given the relatively small pool of potential participants within each institution and the sensitive nature of some disclosures.
