# Supplementary material for: Equity Beyond Entry: A Capability Approach to Understanding Widening Participation in Medical Education
Source: Perspect Med Educ. 2025 Nov 24;14(1):871–81. doi: 10.5334/pme.2071 (PMC12662159; doi:10.5334/pme.2071)
Supplement: Appendix 2. — Interview Guide. [file pme-14-1-2071-s2.pdf]

Experience of Widening Participation Students at Medical School

Widening Participation

"Considering the focus of my research, I would be interested to know what the term widening participation means to you?"

Broad opening

"I wonder if you could you tell me a little about your background and your journey into medical school?"

Probes:

- Do you have any family members or close contacts who are doctors?
- What kind of jobs did the people around you have?
- What sort of educational paths did the other students in your school pursue?
- Did you have support from your family / school teachers / a career advisor?
- How confident did you feel about getting accepted into medical school?
- Do you feel you experienced any hardships or adversity which made it challenging for you?
- What do you think made it possible for you to overcome those barriers?

Experiences of Medical School

"Can you tell me about your experience of medical school?"

Probes:

- Thinking back, was there anything you found surprising or unexpected?
- Are there any ways in which your background made medical school easier or harder for you? (e.g. financial troubles, study skills, travel time, IT resources, academic resources, support…)
- Did your classmates come from similar backgrounds?
- Have you encountered any challenges during medical school?
- What made it possible or harder for you to succeed?
- Did you have any extra responsibilities outside of being a medical student, e.g. carer, working?
- Do you feel you were able to make the most of opportunities available to you?
- How would you describe the support you received from others?

Future Career Building / Postgraduate Training

"Now that finals are out the way, have you thought about moving into the Foundation Programme?"

Probes

- Did you undertake any project work to build towards your foundation application?
- Do you feel you had the same opportunities as your peers to prepare?
- Did you consider applying to the specialised foundation programme?
- Have you thought about your future career path / what specialty you might like?
- In what way do you feel your background will influence your practice as a doctor?

Improvements / Support

“In the context of our discussion today, is there any aspect of your experience that you believe is crucial to understanding the impact of coming from a widening participation background during undergraduate medical education?”

“If medical schools wanted to make it easier for students from your background to thrive, what would need to change?”

Close

“Is there anything else you would like to add to what has been said?”
